# Primo Vascular System in the Lymph Vessel from the Inguinal to the Axillary Nodes

**DOI:** 10.1155/2013/472704

**Published:** 2013-05-21

**Authors:** Seung Hwan Lee, Kyoung-Hee Bae, Geum Ock Kim, Min Ho Nam, Young Bok Choi, Hee-Min Kwon, Yeonhee Ryu, Kwang-Sup Soh

**Affiliations:** ^1^Nano Primo Research Center, Advanced Institute of Convergence Technology, Seoul National University, Suwon 443-270, Republic of Korea; ^2^College of Traditional Chinese Medicine, Science Faculty, University of Technology, Sydney, NSW 2007, Australia; ^3^Yonsei Institute of Convergence Technology, Yonsei University, 162-1 Songdo-dong, Yeonsu-gu, Incheon 406-840, Republic of Korea; ^4^Department of Acupuncture, Korea Institute of Oriental Medicine, Daejeon 305-811, Republic of Korea

## Abstract

The primo vascular system (PVS) in a lymph system was observed mostly in large caliber ducts around the caudal vena cava of rabbits, rats, and mice. This required a severe surgery with laparectomy and massive removal of fat tissues in the abdomen to expose the lymph vessel. In the current brief report, we presented a new method to evade these shortcomings by observing the PVS in a less large caliber duct in the skin, that is, the lymph vessel from the inguinal to the axillary nodes. The Alcian blue injection into the inguinal node revealed the desired primo vessel in the target lymph vessel. This opened a new perspective for the investigation of the lymphatic PVS without severe damage to subject animals and for monitoring of the PVS in a long period of time.

## 1. Introduction

The discovery of the hyaluronan receptor LYVE-1 was a landmark in the research of the lymphatic system which had been one of the least understood systems [1, 2] despite its prominent role in cancer metastasis [3] and inflammation [4]. Another challenge comparable to the development of LYVE-1 is now waiting for solution in the novel threadlike structure called the primo vascular system (PVS) [5] which runs afloat in the lymph flows without attachment to the lymph vessel wall.

Although the presence of the PVS in a lymph vessel (L-PVS) was originally noticed more than fifty years ago [6, 7], no confirmation was made because no method for the observation was described in the literature. Only recently L-PVS was extensively observed by developing several methods with staining dyes: in the case of rabbits with the dye Janus green B [8], Alcian blue [9, 10], and optical method [11], in the case of rats with magnetic fluorescence nanoparticles [12], and Alcian blue [13], and in the case of mice with a Prox1-GFP transgenic mouse without injection of dyes [14]. 

The possible significant role of the PVS was suggested by the observation of immune cells abundant in the L-PVS with population ratio as mast cell (20%), eosinophil (16%), neutrophil (15%), lymphocyte (1%), immature cells (3%), and chromatin cell (0.3%) [13]. The presence of hyaluronic acids and the hormone adrenalin and nor adrenalin in PVS was confirmed in this work as claimed earlier by Kim [7, 13, 15].

 Until the present work, the PVS in the lymph system was observed mostly in the large caliber lymph vessels running along the caudal vena cava from the lumbar node to the mesenteric node near the kidney [8–13]. The only exception was the PVS in the rodent thoracic ducts [14]. Another rare case was the primo vessel in a lymph vessel which came from a tumor that was xenografted in the ventral skin of a mouse [16]. This case is a reinforcing example of the conjectured role of the PVS in cancer metastasis that was raised in an earlier work [17].

 In the current work, the PVS in the lymph vessel from the inguinal to the axillary lymph nodes in the abdominal skin was first observed by injecting Alcian blue into the inguinal lymph node. It was a big step forward in the sense that it became possible to observe the L-PVS without laparectomy and moving the intestines to sideways to expose the lymph vessels. It also required removal of the fat tissues around the lymph vessel. This surgery operation was a severe damage to the physiological condition of the subject animal. The current work will lead to a less damaging observation of the L-PVS without severe surgery for studying its functional aspects and future progress toward monitoring system of L-PVS for a long period of time. The monitoring would open to the physiological understanding of the periodic generation of cells in the primo nodes as claimed by Kim [7].

## 2. Materials and Methods

### 2.1. Animals

Five male Sprague-Dawley (SD) rats (7 to 9 weeks) were obtained from DooYeol Biotech (Seoul, Republic of Korea). The animals were housed in constant temperature and humidity conditions (23°C, relative humidity 60%) with a 12/12 hour light/dark cycles and were provided water and commercial rat chow *ad libitum*. The procedures involving the animals and their care were in full compliance with current international laws and policies (*Guide for the Case and Use of Laboratory Animals*, National Academy Press, 1996), which were approved by the Institutional Ethics Committee of the Advanced Institute of Convergence Technology (approval number is WJIACUC20130212-1-07).

### 2.2. *In Vivo* Operation and Visualization of the Primo Vascular System

The rats were anesthetized by intramuscular injection of a regimen consisting of 1.5 g/kg of urethane and 20 mg/kg of xylazine. An incision of the subcutaneous layer of the skin along the linea alba was performed with surgical scissors, and the incised skin was bent back for exposure of the target inguinal lymph nodes.

The staining dye, 1% Alcian blue (A5268, Sigma-Aldrich, St. Louis, MO, USA) solution in phosphate-buffered saline (PBS, pH 7.4), was prepared and was filtered by using a 0.22 *μ*m membrane filter (Merck Millipore, Darmstadt, Germany) with a syringe (BD, Franklin Lakes, NJ, USA). After incising the subcutaneous layer of rats along the linea alba, Alcian blue solution, preheated to 37°C in a warm bath, was injected into a inguinal lymph node. In order to help the dyeing agent to be thoroughly washed out, it is needed to promote the natural circulation of the lymph fluid. To do that, preventing a decrease of body temperature is necessary. Therefore, the rats were covered with paper tissue and kept on the enough rat bedding.

After three hours of dye injections, the inguinal lymph node and the lymph vessel between inguinal lymph node and axillary lymph node of the rat were exposed to observe with removing the fat tissue surrounding them. For better observation, bleeding should be minimized during operation. All procedures of observation and operation were performed under a stereomicroscope (SZX12, Olympus, Japan). After observing the stained primo vessels in the lymph vessel, the rats were sacrificed by intracardiac injection of urethane 1 mL. The whole sacrificed rats are needed to be fixed with 10% neutral buffered formalin (NBF) solution at 4°C for 24 hours. 

The lymph vessels including stained primo vessel were collected from the fixed rat under the stereomicroscope. For morphological analysis, the primo vessel was extracted from the lymph vessel; for histological analysis and immunofluorescence, the whole lymph vessel including primo vessel was prepared.

### 2.3. Analysis of the Primo Vascular System

#### 2.3.1. Morphological Analysis

For the staining of nuclei and f-actins in the cells, 4′,6-diamidino-2-phenylindole (DAPI) and phalloidin were applied, respectively. The specimen was stained with 300 nM DAPI (D1306, Invitrogen, MO, USA) solution for 20 minutes and 6.6 *μ*M phalloidin 488 (A12379, Invitrogen, MO, USA) solution for 30 minutes. After washing, the specimen was covered with mounting solution. The stained specimen was investigated under a confocal laser scanning microscope (CLSM; C1 plus, Nikon, Japan). 

#### 2.3.2. Histological Analysis and Immunofluorescence

In order to examine the structure of the primo vessel, the sample was cross-sectioned, and hematoxylin and eosin (H&E) staining was performed. The specimen was first embedded in the gel matrix (Tissue-Tek OCT Compound, Sakura Finetek, Japan) and was kept in −80°C for several hours. The sample was sectioned in the coronal direction of the PV at a 5 *μ*m thickness using a cryotome (CM1800, Leica, Germany), and a routine H&E staining was followed. The stained specimen was investigated under a phase contrast microscope (BX51, Olympus, Japan).

With the understanding that primo vessel has EMP-3 positive cells in its outermost tissue, immunofluorescence with EMP-3 antibody (H-130 and W-14, Santa Cruz, CA, USA) as a primary antibody and Alexa Fluor 488 (A11034, Invitrogen, MO, USA) as a secondary antibody was performed. After fixation and permeabilization with acetone, the cross-sectioned specimens were applied CAS block (008120, Invitrogen, MO, USA) and placed in a humid box for 2 hours at room temperature. The primary antibody, diluted into 1 : 200 in blocking agent, was applied overnight at 4°C, and then the secondary antibody, diluted into 1 : 500 in PBS, was applied for 2 hours at room temperature. DAPI was used for counterstaining. The specimen was investigated under a fluorescent microscope (BX51, Olympus, Japan).

## 3. Results

**Figure 1:**
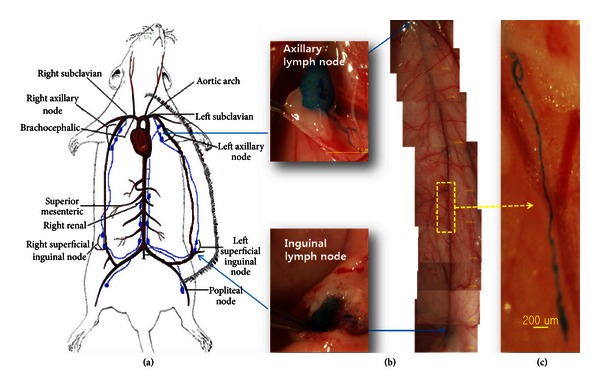
Visualization of the primo vascular system in the skin lymph vessel between inguinal lymph node and axillary lymph node. (a) A schematic diagram of large caliber lymphatic system in a rat. (b) Axillary and inguinal lymph nodes and the lymph vessel between them. Alcian blue was injected into the inguinal lymph node and it flowed up to the axillary lymph. (c) Magnified view of the rectangular area in (b). A primo vessel stained by Alcian blue was visualized in the lymph vessel (the fuzzy milky white tissue).

The PVSs in previous researches were observed in the large caliber lymph vessel and its branches between the lumbar and the mesenteric nodes and in the thoracic duct as illustrated in Figure 1(a). In the current work, the PVS was found in the skin lymph vessel from the inguinal to the axillary nodes (Figures 1(a) and 1(b)). In Figure 1(c), the inguinal-axial (I-A) lymph vessel was the milky white tissue around the thin blue curve in it. The blue color was due to the Alcian blue absorbed by the primo vessel. The skin with the I-A lymph vessel was removed from the rat, fixed with NBF, and put on a slide. It was noticeable that the primo vessel shown in Figure 1(c) had coil-shaped regions with both upper and lower ends. This was because the primo vessel was an elastic thread-like structure. This phenomenon was noticed in earlier works on other kinds of PVS than the L-PVS.

**Figure 2:**
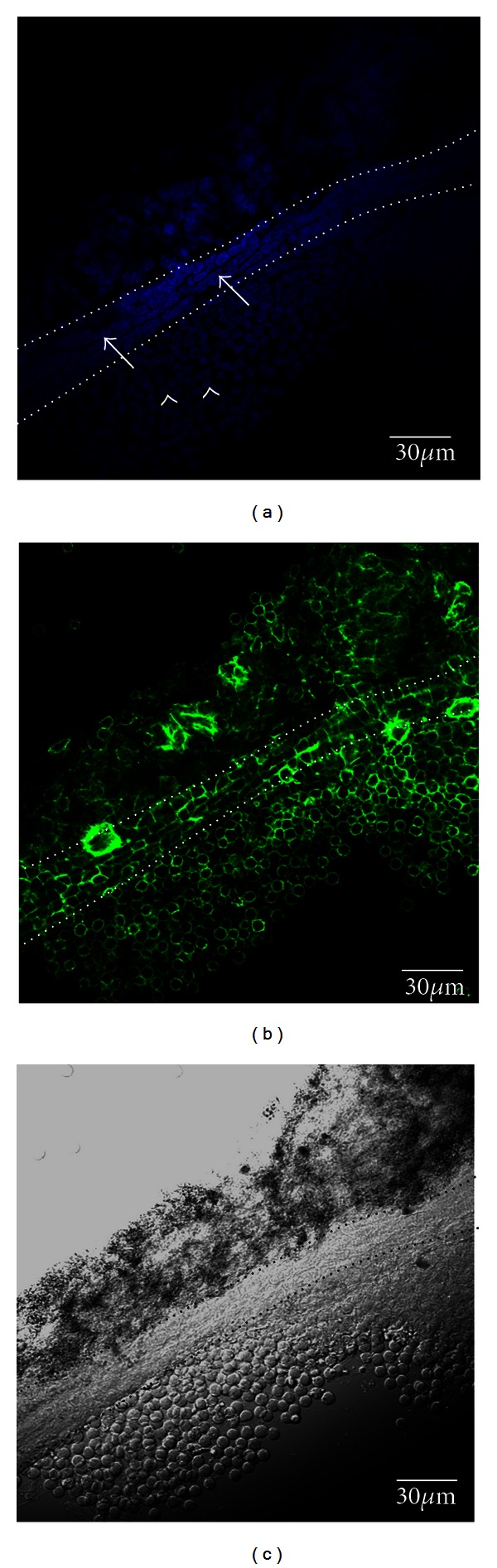
Confocal laser scanning microscope images of the primo vessel extracted from the lymph vessel. (a) DAPI signal image of the primo vessel (dotted line). Notice some rod-shaped nuclei (arrows) which were clearly differentiated from the round ones (arrowheads) of lymphocytes. (b) Phalloidin signal image of the primo vessel. The pattern of the f-actins was different from that of lymphocytes. (c) Differential interference contrast image of the primo vessel. The PVS-region was clearly distinguishable from the aggregate parts even without any fluorescence.

Figure 2 showed the differential interference image and the fluorescence images of a primo vessel specimen that were taken out from the lymph vessel. The hallmark of a primo vessel is the distribution and shape and length of nuclei. Indeed, Figure 2(a) showed that the rod-shaped nuclei stained with DAPI were aligned longitudinally parallel to the primo vessel. The lengths of the nuclei were between 10 and 20 *μ*m as expected [6]. The phalloidin staining signals of the f-actins in the cells (Figure 2(b)) were also aligned along the vessel direction. These two features were in good agreement with previous PVS works [18]. The differential interference contrast image also clearly distinguished the PVS from the aggregated lymphocytes (Figure 2(c)).

**Figure 3:**
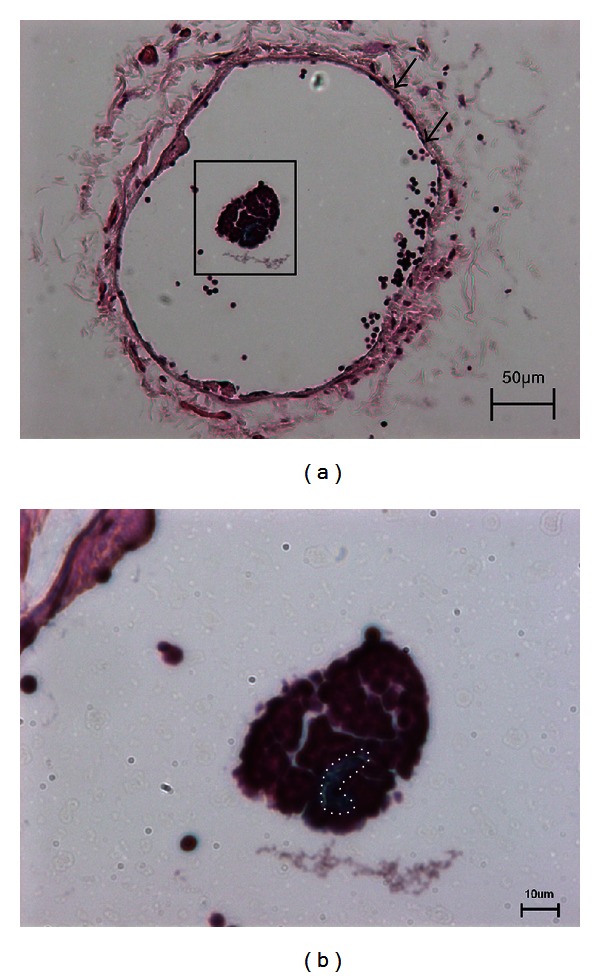
H&E stained primo vessel in the lymph vessel. (a) A primo vessel (rectangle) was inside the lymph vessel (arrows). (b) Magnified image of the rectangle in (a). The genuine primo vessel (dotted line) stained with Alcian blue was surrounded with aggregated lymphocytes. The diameter of the genuine primo vessel was only about 20 *μ*m. However, it was difficult to define the boundary of the PVS. The data in Table 1 was over estimated due to the aggregated region.

**Figure 4:**
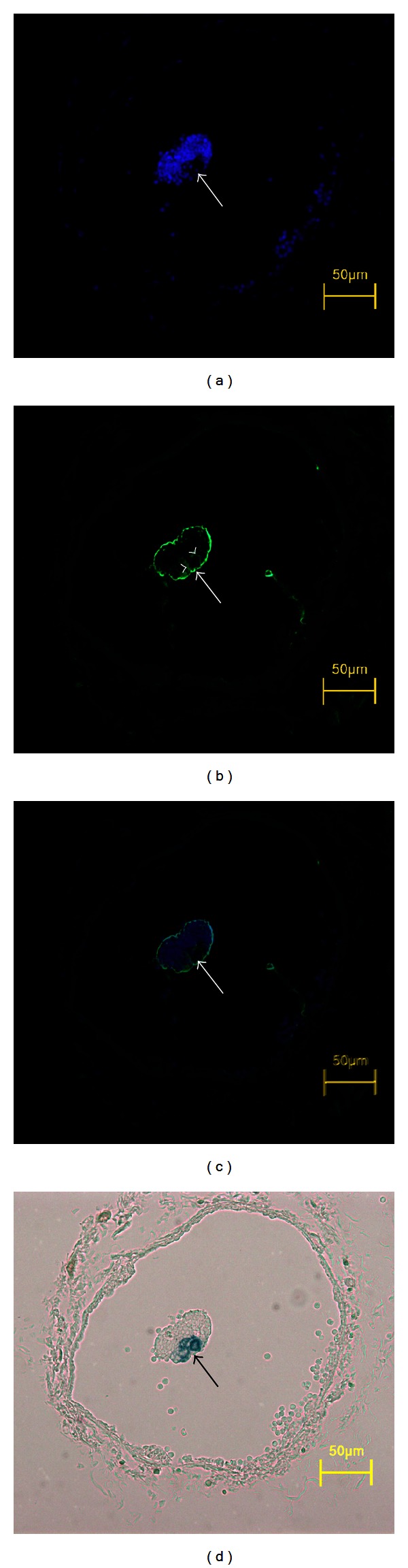
Immunofluorescence with EMP-3 of primo vessel in the lymph vessel. (a) DAPI signal image of the cross-sectioned primo vessel. Notice that only few nuclei were distributed in the region of the genuine primo vessel (arrow). This was in agreement with the structure of primo vessel with bundles of fibers outside the endothelial cells. (b) EMP-3 signal image of the cross-sectioned primo vessel. EMP-3 was positively reacted with the surrounding cells of the genuine primo vessel. These cells were epithelial cells at the boundary of the primo vessel. The very strong signals at the larger boundary of the aggregated lymphocytes were not understood yet. (c) Merged image of (a) and (b). (d) Phase contrast image of the cross-sectioned primo vessel. The genuine primo vessel was stained with Alcian blue, whereas the other part, aggregation of lymphocytes, was not.

The basic histological examination of the primo vessel was done with H&E, and the immunofluorescence with EMP-3 as shown in Figures 3 and 4. The H&E image and its magnified one (Figures 3(a) and 3(b)) showed the aggregated nuclei of the lymphocytes around the Alcian blue stained region with very few nuclei. This lack of nuclei was a desired result showing the fiber bundle structure of the primo vessel with few endothelial cells inside. However, the boundary between the PVS and gathered lymphocytes was not delineable.

The EMP-3 signal around the primo vessel region provided the desired boundary information because this signal was positively expressed by the epithelial cells of the PVS as was reported earlier [13].  Figure 4(a) depicted the distribution of nuclei stained blue with DAPI, Figure 4(b) the EMP-3 signals, and Figure 4(c) the combined images. There were only few nuclei in the primo vessel, and the definite signal of EMP-3 was seen at the boundary of the primo vessel. The phase contrast image also demonstrated the primo vessel stained with Alcian blue and covered with gathered lymphocytes. The large strong EMP-3 signal around the boundary of the gathered lymphocytes was not PVS-related, and its appearance was not yet understood. The basic morphological data of the lymph vessel, the primo vessel, and the primo nodes were summarized in Table 1.

## 4. Discussion

 Primo vessels in the lymph ducts from the inguinal to the axillary nodes were found and identified with histological features. It was the first case of the L-PVS that was observed in the lymph system in the skin. This was significant because the PVS was present not only in the large caliber lymph ducts, but also in less large lymph vessels. Expected physiological functions of the PVS were related to immune functions as the PVS contains abundant immune cells [13] and to regeneration as it has hematopoietic stem cells [19]. Another important function is cancer metastasis via PVS as the migration of cancer cells through a primo vessel was observed [20]. 

The immunohistochemistry with EMP-3 was in agreement with the earlier work [13] and provided a proof that the PVS in the current work was not an artifact induced by aggregates of materials from lymph fluid. This was because the EMP-3 expression of epithelial cells was in the Alcian blue stained part of the threadlike structure. The signals from the aggregates of lymphocytes were also very strong only at the boundaries. In general, separated lymphocytes were not positive with EMP-3. This strong signal was a kind of exceptional expressions which are not understood at the moment and requires, further investigation.

The basic histology with H&E also supported the threadlike specimen as an intrinsic bundle structure of collagens with endothelial cells as described by Kim [6, 7]. It demonstrated clearly that the aggregates sometimes thickly covered the primo vessel and it was difficult to delineate the boundary between the primo vessel, and the aggregates. It is highly desirable to develop a technique to obtain a pure primo vessel. 

 About the possible relevance with respect to acupuncture, it is difficult to prove whether it is connected to acupuncture points or meridians at the moment. It was recently shown that the PVS on the surfaces of internal organs is not related to acupuncture [21]. However, directly related subclasses of the PVS are those in the skin or along the outside wall of blood vessels and nerves, which have not yet been observed. Therefore, the connection with traditional Chinese medicine of the PVS needs to be investigated with the extravascular type of PVS.

## Figures and Tables

**Table 1:** Gross anatomy of the primo vascular system in the lymph vessel between inguinal and axillary lymph nodes.

Subject number	Age (week)	Length of I-A lymph vessel (cm)	Diameter of I-A lymph vessel (*μ*m)	Diameter of primo vessel (*μ*m)	Number of primo node (number)	Size of primo nodes (short axis (*μ*m) × long axis (*μ*m))
1	9 w	9.1	415	56	8	86 × 261
2	9 w	9.0	390	56	5	75 × 212
3	9 w	8.7	320	37	3	39 × 150
4	8 w	7.8	334	33	3	36 × 101
5	7 w	6.6	387	58	6	107 × 239
Average ± S.D	—	8.2 ± 1.1	369 ± 40	48 ± 12	5 ± 2	69 ± 31 × 193 ± 66
